# Epidemiology of Chagas Disease in the United States of America: A Short Review and Some Comments

**DOI:** 10.3390/pathogens14010024

**Published:** 2025-01-01

**Authors:** Stephen A. Klotz

**Affiliations:** Division of Infectious Diseases, University of Arizona, 5701 North Campbell Ave., Tucson, AZ 85719,USA; sklotz@u.arizona.edu

**Keywords:** Chagas disease, *Trypanosoma cruzi*, kissing bugs, epidemiology

## Abstract

The epidemiology of Chagas disease in humans has markedly changed within the past several decades in the United States of America. This report discusses autochthonous cases of Chagas disease as well as disease in immigrants from Latin American countries. Suggestions for epidemiology research and medical care are discussed given the evolving epidemiology of the disease in the United States of America.

The epidemiology of Chagas Disease (CD) in the United States of America (USA) has never been more important than at present. Accurate data is needed to carefully plan for and obtain the medical resources required to identify and treat people with CD in the USA. The influx of millions of immigrants within the past several decades, some of whom are infected with *Trypanosoma cruzi* (Chagas, 1909), has changed the nature of CD in this country. In addition, epidemiologic studies will also provide a direction for future research of CD in the USA. In this report, the discussion will be confined to the epidemiology of CD in humans, only referring to the enzootic relationships among mammals and kissing bugs as needed to knit together the complex life cycle of the pathogen.

*Trypanosoma cruzi* is found within three major reservoirs (hosts) in the USA. The first reservoir is the many species of small-to-medium-sized burrowing and nesting wild mammals. These animals, in turn, are fed upon by 11 species of hematophagous insects known as “kissing bugs” in the USA (family Reduviidae, subfamily Triatominae). Kissing bugs are scattered over the lower 2/3 tier of states and constitute the second reservoir. The third reservoir of *T. cruzi* is humans. Some hosts among mammals and humans are susceptible to disease once infected. At present, the human reservoir in the USA is preponderantly composed of infected immigrants from Latin American countries endemic for CD (these individuals may number in the thousands, perhaps hundreds of thousands). In addition, there is a smaller number of autochthonous cases of CD, numbering from fifty to a hundred [1]. Kissing bugs are the link or vector between the mammals and humans, with infection occurring via fecal contamination either orally or percutaneously. In addition, humans, through blood transfusions, transplants, and childbirth, can be a source of infection for other humans.

## 1. Chagas Disease in the USA

The first description of *T. cruzi* in the USA was provided by Kofoid and McCulloch, who reported the pathogen in the blood of wood rats (*Neotoma* spp.) in California in 1916 [2]. Unlike Chagas’ discovery in Brazil a decade earlier, where he described the pathogen, insect vector, and human disease, all within several years [3,4,5], the first autochthonous human case of CD in the USA was not reported until 1955 [6]. This lapse of 39 years between the first description of disease in a wood rat and disease in a human in the USA provides an interesting contrast between the discovery of the pathogen in the savannah of Brazil and finding it in the xeric landscape of California. Geography is a contributing factor to differences in the incidence of CD between Latin America and the southern border of the USA. However, the US–Mexico border is a barrier in name only between the two countries of different cultures, different housing stock, different kissing bug species, and differing human genetic pools. The border allows for the almost free movement back and forth of mammals, insects, and humans, as well as their parasites. Although Mexico and the USA share three competent vectors of CD at the border, including *T. protracta* (Uhler)*, T. recurva* (Stahl, 1868), and *T. rubida* (Uhler, 1894) [7,8,9], the autochthonous incidence of human CD is vastly different between the two countries. It has recently been estimated that 1,182,286 cases of CD are present in Mexico (0.96% of the population) [10]. Another species, *T. longipes* (Barber, 1937), was reported by Wood to occur in Arizona [11]; however, this species is considered synonymous to *T. recurva* [12].

The natural history of kissing bugs and their interaction with humans has been studied for these three species over the past 15 years in Arizona. We concluded that because there is little or no domiciliation of the bugs in the USA and, in addition, taking into account the costive defecatory patterns of Arizona bugs, infecting humans with CD in the USA seems unlikely [13].

## 2. Latin American Immigrants to the USA and Chagas Disease

The arrival of Chagas disease into the USA through infected Central American immigrants was reported in 1987 [14]. The authors studied 205 Salvadorian and Nicaraguan immigrants living in the Washington DC area and found that 10 (4.9%) had positive serologies for *T. cruzi*. Three individuals of six were also positive by xenodiagnoses. In concluding the paper, the authors stated “The general quality of housing and the absence of reduviid species that readily become domiciliary are the most likely explanations for the rarity of *T. cruzi* transmission to humans in the United States. The presence of additional numbers of *T. cruzi*-infected immigrants will not change this situation [15].”. The first sentence explains a great deal about the rarity of autochthonous human cases in the USA, viz. that housing stock amongst the poor in Latin American countries allowed reduviids to domiciliate, thus giving them unrestricted access to human residents. This does not occur in the USA [13]. However, the concluding sentence (quoted above) is arguable, given the magnitude of immigration into the USA. Immigrants have brought along many new isolates (“near clades”) [14] of *T. cruzi* that have the potential to change the behavior of the kissing bugs and parasites in the USA when acquired by indigenous kissing bugs [16,17,18,19].

Schmunis in 2007 attempted to quantitate the numbers of infected immigrants, estimating there were 56,028 to 357,205 infected individuals among 7.2 million legal immigrants to the USA from 1981 to 2005 [20]. Shortly thereafter, Bern and Montgomery published a figure of 300,167 cases of CD among immigrants to the USA [21]. This is the most cited report regarding infected immigrants. Lacking real numbers concerning cases of CD, these reports and others use aggregate prevalence data for each Latin American country from which the immigrants originated to determine (estimate) the number of cases of CD. In addition, the number of infections that are projected to progress to either cardiovascular or gastrointestinal syndromes are an estimate as well. These estimates usually range from 20 to 40% of those infected. The difficulties (inaccuracies) involving the use of estimates were noted and appreciated in the original 2009 publication. Unfortunately, in the absence of actual evidence (i.e., proven cases), not the fault of the authors, these estimates are often treated as fact by readers.

A problem plaguing all the epidemiological studies of CD is not only the use of estimations but that the data are derived mainly from asymptomatic people who have indeterminate or chronic infection, and there is no possibility of accurately determining when or where infection occurred. This fact makes determining whether positive serologies reported in the USA represent autochthonous infections or imported infections very difficult to determine. For example, the original case descriptions in the USA, without exception, described individuals with an acute illness accompanied by a peripheral blood smear demonstrating *T. cruzi* in the blood. Interestingly, of the first seven documented CD infections in the USA, four were acute CD in children or infants [22]. An example of the difficulty is a recent paper reporting 18 autochthonous cases of CD in Texas [23]. However, the border area between Mexico and Texas was intensively studied in the past and no cases of CD were described from 1909 participants living in “appalling” conditions in 1949 [24]. The possibility exists that these “new cases” may reflect the changing ethnic composition of the state rather than increased numbers of autochthonous cases of CD. However, as stated before, there is no way to accurately determine the date and location of the acquisition of CD by the use of serology unless other data are provided.

## 3. Given the Present Status of CD in the USA and What We Know Now, What Should Be Done?

Screening immigrants is a priority, but in addition, screening citizens identified as “at risk” by local authorities will be important as well. Hence, it is urgent that we identify one test or a battery of tests to serve as the “Gold Standard” for the serological diagnosis of CD. Choosing a gold standard that would be adopted countrywide would facilitate the identification of infected individuals to begin the process of screening [25]. Since testing at the Centers for Disease Control (CDC) is currently regarded as the “Gold Standard” in the USA, perhaps scaling up testing at the CDC would be the most efficacious approach. (Their requirements for a positive serology require that at least two of three different tests be positive.) It would be prudent if female immigrants from CD-endemic countries under 40 years of age and immigrant males under 20 years of age undergo serological testing for CD. The risk for delivering an infected newborn would be identified and treatment offered when needed. It is possible that other measures may be required in the future as the Chagas epidemic unfolds in the USA among permissive kissing bug species and wild mammals.

Since *T. cruzi* is mostly a clonal pathogen [26], it is important to determine what effect the introduction of many new clones of *T. cruzi* into the USA will have upon native kissing bugs as well as *T. cruzi* itself. Furthermore, what effect, if any, new clones of *T. cruzi* will have upon the clinical presentation of CD if acquired autochthonously should be closely watched.

A list of autochthonous USA cases should be compiled with pertinent clinical and historical findings and patients revisited over time to determine the outcome of the infection. For example, do the percentages of cardiac and gastrointestinal complications known to occur in Latin America predict what will occur in autochthonous cases in the USA? Autochthonous cases (treated and untreated) should be revisited regularly to determine how often serologies turn negative. If and when serologies turn negative, does this mean it is cured [27]?

Investigators should adopt stricter criteria for autochthonous cases in the USA. Individuals should not be counted as autochthonous if they spent two weeks or less in an endemic country, since an individual could, and undoubtedly has, visited an endemic area in Latin America and been infected on day one of their visit. Relying on some arbitrary timeline is tenuous at best and individuals with a travel history to endemic countries should be placed in a separate category. This also applies to identifying cases as autochthonous based upon the possession of European surnames, as was done in one report [28]. Problems of categorization will continue to arise because Chagas’ infection when acquired is overwhelmingly asymptomatic. Unfortunately, a positive serology cannot tell the investigator when infection occurred, and this is a critical piece of information when deciding whether an infection is autochthonous or not.

Although eradicating kissing bugs in Latin America has been effective in reducing CD, there is probably little to be gained by trying to interrupt vector-borne disease in the USA, as domiciliation by the bugs is uncommon and home entry sporadic [29]. However, educating the public about the risk of transmission of *T. cruzi* to humans by kissing bugs as well as by mammals infected with *T. cruzi* is important. Home hygiene is a particularly important feature in southwestern USA, where home intrusions by kissing bugs occur yearly in great numbers.

## Data Availability

Not applicable.
